# Community based system dynamic as an approach for understanding and acting on messy problems: a case study for global mental health intervention in Afghanistan

**DOI:** 10.1186/s13031-016-0089-2

**Published:** 2016-11-02

**Authors:** Jean-Francois Trani, Ellis Ballard, Parul Bakhshi, Peter Hovmand

**Affiliations:** 1 Brown School of Social Work, Washington University in St Louis, 1 Brookings Drive, St Louis, MO 63130 USA; 2 Program in Occupational Therapy, School of Medicine, Washington University in St Louis, St Louis, USA

**Keywords:** Afghanistan, Causal loop diagram, Community based system dynamics, Complex problems, Development intervention, Mental health

## Abstract

**Background:**

Afghanistan lacks suitable specialized mental healthcare services despite high prevalence of severe mental health disorders which are aggravated by the conflict and numerous daily stressors. Recent studies have shown that Afghans with mental illness are not only deprived of care but are vulnerable in many other ways. Innovative participatory approaches to the design of mental healthcare policies and programs are needed in such challenging context.

**Methods:**

We employed community based system dynamics to examine interactions between multiple factors and actors to examine the problem of persistently low service utilization for people with mental illness. Group model building sessions, designed based on a series of scripts and led by three facilitators, took place with NGO staff members in Mazar-I-Sharif in July 2014 and in Kabul in February 2015.

**Results:**

We identified major feedback loops that constitute a hypothesis of how system components interact to generate a persistently low rate of service utilization by people with mental illness. In particular, we found that the interaction of the combined burdens of poverty and cost of treatment interact with cultural and social stigmatizing beliefs, in the context of limited clinical or other treatment support, to perpetuate low access to care for people with mental disorders. These findings indicate that the introduction of mental healthcare services alone will not be sufficient to meaningfully improve the condition of individuals with mental illness if community stigma and poverty are not addressed concurrently.

**Conclusions:**

Our model highlights important factors that prevent persons with mental illness from accessing services. Our study demonstrates that group model building methods using community based system dynamics can provide an effective tool to elicit a common vision on a complex problem and identify shared potential strategies for intervention in a development and global health context. Its strength and originality is the leadership role played by the actors embedded within the system in describing the complex problem and suggesting interventions.

## Background

A recent study has shown that the global burden of mental illness has been systematically underestimated. Revised estimates show that mental illness accounts for 32 · 4 % of years lived with disability (YLDs), ranking mental illness first in terms of YLDs [1]. Despite a growing body of empirical evidence showing the considerable personal and socioeconomic impact of this burden, existing treatment options for persons with mental illness are limited [2]. It becomes increasingly clear that is possible to develop mental health treatment programmes in low income settings. Some NGOs have developed programs to address the mental health needs of populations in post-emergency settings such as the NGO HealthNet TPO in Afghanistan, Burundi or International Assistance Mission in Afghanistan [3, 4]. In the province of Aceh in Indonesia, in the post tsunami period, the Ministry of Health and the World Health Organization set up a community-based mental health system integrating mental health services within primary healthcare facilities, with secondary mental care available at the district general hospitals and tertiary and specialized care provided at the provincial general hospitals level [3]. More generally, the the World Health Organization (WHO) Mental Health Gap Action Plan (mhGAP) provide guidelines for the provision of drugs and psychosocial interventions and has informed several programs aiming primarily at integrating mental health into primary care in Low and Middle Income Countries (LMICs) [5–7]. Many other innovative initiatives such as the PRogramme for Improving Mental health carE (PRIME) or Africa Focus on Intervention Research for Mental health (AFFIRM) and Emerging Mental health systems in low and middle-income countries (EMERALD) have been generated evidence on the implementation, capacity development and scaling up of mental health packages aiming at narrowing the treatment gap for mental disorders [8–10].

Yet the reach of these programs remains limited and many persons with mental illness (PMI) remain in need of mental healthcare services. Complex and interacting supply-side barriers of resource availability, costs of treatment, and logistical challenges to sustaining services, as well as demand side factors such as out-of-pocket expenditures, long term chronic needs and social factors such as stigma around mental illness, acceptability of the setting in which treatment is delivered, and lack of family participation in treatment and sensitization efforts have been shown to be major obstacles to widespread access to mental health services [11–17]. In public health these complex and seemingly intractable challenges are variously referred to as “wicked problems” [18, 19] or “messy problems” [20]. Addressing such barriers in low-income settings would require an integrated approach that involves people with mental illness themselves, their families and communities, as well as building local capacity in existing healthcare facilities [13]. Another perspective argues that dominant approaches to promoting health fail to account for the diversity of the “Long Tail” of vulnerable populations – diverse social groups with specific socioeconomic characteristics that have various exposure to fundamental health risks, resulting in a failure to reach the most marginalized [21], among whom the burden of morbidity and mortality is greatest [22–25]. From both perspectives, the challenge often comes down to the inadequacy of conventional analytic and planning tools to capture the complexity of problems operating at multiple levels and with diverse stakeholder perspectives and contexts. The broad framework of “participation” in global health and development efforts has been variously embraced [26, 27] and critiqued [28, 29] as a solution to engaging with diverse local needs. Yet there is little uptake of approaches to designing policies and programs that engage with complexity, respond to the needs and promote the capabilities of the most vulnerable [30], and provide concrete steps for action.

Mental healthcare in countries in conflict represents a particularly ‘messy’ problem that, despite significant discussion among scholars and international development actors [31], has not been prioritized to develop widespread, effective and well-funded intervention, particularly in low income countries and fragile contexts [32]. Limited availability of data in low income countries [33–35], wide variation in social and cultural definitions and interpretations of mental disorder [36, 37], and limited evidence about the efficacy of intervention approaches [38, 39] all pose barriers to progress.

**Fig. 1:**
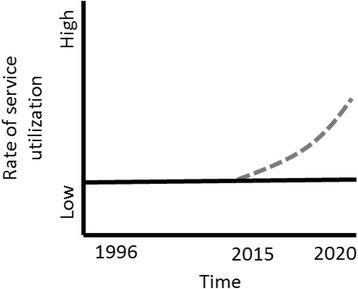
Reference mode: Rate of service utilization by people with mental disorders

In Afghanistan recent studies have reported high prevalence of various mental health disorders linked to the conflict and various psychosocial stressors associated with poverty, loss of employment, drug abuse and traumatizing events [40–42]. Despite important initiatives, the current lack of mental healthcare services is a considerable challenge. To date, Afghanistan lacks widespread access to mental health services despite successful pilot interventions in the province of Nangarhar [4, 43, 44] and the integration and recent scaling up of psychosocial models of treatment into the basic package of health services (health posts, health centers and district hospitals) [44–46]. Moreover, the prioritization of mental health support in community-based interventions such as Community Based Rehabilitation (CBR) [47, 48], has not translated into widespread effective mental health programs in Afghanistan being delievered through the CBR platform. The persistent low utilization of healthcare services by persons with mental disorders represents a dynamic problem (Fig. 1) that challenges both policy makers and program implementers.

Community based system dynamics (CBSD) represents a novel approach that holds promise for problem analysis and policy design. Like other participatory approaches such as Theory of Change (ToC) or Participatory Action Research (PAR) used to address public health issues [49–51], CBSD engages stakeholders who are embedded in a system to examine complex problems [52]. CBSD highlights the feedback within systems, and examines dynamic change in system behavior over time, as well as nonlinear relationships, allowing for explicit engagement with causal mechanisms in complex problems. CBSD provides a structured process and forum for diverse stakeholders to identify issues and prioritize intervention through the language of systems, and to support the development of stakeholders’ capacity to engage with practical problem-solving [20, 53].

In the present study, we report on a CBSD-informed Group Model Building (GMB) workshop to consider how an Afghan community based rehabilitation program might effectively expand its interventions to cover the needs of people with mental illness.

This paper examines the dynamics of mental health service seeking and capacity for supporting people with mental illness from the perspective of a CBR program operating in Afghanistan. It proposes insights into ways to enhance access to mental health services for people with mental illness (PMI).

## Methods

### Setting

The study was carried out with staff of a CBR program providing services for persons with disabilities in 13 provinces of Northeastern Afghanistan.

### Study design and participants

We initiated a series of Group Model Building sessions with Community-Based Rehabilitation workers (CBRW) and CBR team leaders from a large international organization operating in the northern and eastern regions of Afghanistan. The purpose of the sessions was to investigate questions arising from initial findings of a 3-year impact evaluation research study. Initial GMB sessions were held over in June 2014 in Mazar-e-Sharif, Balkh, Afghanistan, and follow-up sessions were conducted in Kabul, Afghanistan in February 2015. The initial sessions were conducted with three males and three females community based rehabilitation workers from the Mazar-e-Sharif region. Sessions were also conducted with four males CBR workers from Jalalabad to triangulate findings of the first sessions. The follow-up sessions consisted of two male and two female research officers with experience in both CBR and research methods (Table 1). These four participants in the follow-up sessions were from Mazar-e-Sharif, Taloqan, Ghazni and Jalalabad, four regional program offices of our partner NGO.

**Table 1:** Description of participants in the GMB sessions

Session location and date	Participants number and profile	Gender
Sessions 1 and 2 June 2014	6 participants, community based rehabilitation workers from Mazar I Sharif	3 females and 3 males
Sessions 3 and 4 Mazar I Sharif June 2014	4 participants, community based rehabilitation workers from Jalalabad	4 males
Session 5,6 and 7 February 2015	4 research officers from Taloqan, Mazar I Sharif, Ghazni and Jalalabad	2 females and 2 males

Sessions were planned based on a series of scripts adapted from *Scriptapedia* a manual composed of structured group model building activities [54]*,* and were led by a team consisting of Afghan NGO staff members and of three international researchers as facilitators. Sessions included a series of scripts (Table 2) designed to explore the interactions and interdependencies between factors affecting participation of people with severe and disabling forms of mental disorder in CBR activities, and to develop a common model of the complex local dynamics and explore possibilities for intervention to provide care to PMI. In particular, session particpants described the existing relational dynamics among the set of factors identified by constructing *causal loop diagrams (CLDs)*.

**Table 2:** Group model building session agenda and description of “Scripts”

Session 1: June 2014, Mazar-e-Sharif, Balkh, Afghanistan: The introductory session took place over the course of an afternoon in Mazar-e-Sharif to explore the interacting factors that may explain low participation of people with mental illness in CBR programs	
Activity	Description
Introduction to systems, Defining Terms	Introduction of the approach of community based system dynamics
	Defining concepts – “What do we mean when we say ‘Mental Illness’?
Variable Elicitation	Participants nominated factors or variables that responded to the prompt: “What causes Rawani to receive or not receive rehabilitation services”
Stars	Participants prioritized the most relevant and impactful variables produced in the previous variable elicitation activity.
CLD Elaboration	Based on the priority variables emerging from the stars exercise the facilitators led participants through an exercise to develop a causal loop diagram describing causal structure and feedback relationships.
Model Review	At the end of the CLD Elaboration activity, facilitators led participants through a structured exercise to restate common definitions established for Rawani and identify important feedback loops and exogenous variables. A later discussion revisited the model to identify preliminary points for potential intervention by CBR program activities.
Session 2: Febbruary 2015, Kabul, Afghanistan: This session took place over two meetings in three days using a series of models to explore the dynamics of social inclusion of people with mental illness and articulate potential strategies for programmatic intervention	
Session 2.1	
Introduction	Participants had previously been oriented to group model building through a research methods seminar. An opening discussion examined the question “What distinguishes Rawani vs Diwana”? Participants shared examples of scenarios in which a family member or community member might be considered Rawani or Diwana, and prompting facilitators and fellow participants probed to draw contextual distinctions between the two concepts.
Variable Elicitation	Participants nominated variables based on the prompt “What would be conditions for including people with ‘psychological problems’ in CBR activities?”.
Priorities	Each participant was asked to vote for the three most important variables in the inclusion of people with “psychological problems” in CBR activities.
CLD Elaboration	The highest rated variables were used to seed the structure for elaborating a causal loop diagram on sheets of chart paper that had been taped together. Participants nominated causal links, with pauses to discuss the specific assumptions of causality or negotiate definitions of terms as questions emerged.
Model Review	At the close of the first day of the session, facilitators identified major themes that emerged from the session, highlighted major feedback loops from the session, and discussed potential areas for further development or exploration.
Session 2.2	
Revisiting the CLD Model	The first day’s model, was posted beside blank chart paper, and core structure for the second day model building as identified on the old model and redrawn on the new model paper. Questions about translation or recopying were discussed.
CLD Elaboration	New causal structure was built onto the seed structure identified in the previous activity.
Model Review	At the end of the session major feedback loops, themes, and remaining questions or exogenous variables were identified. A further discussion explored potential points for programmatic or policy intervention revealed by the model.

### Analysis

Preliminary analysis occured during the process of elaborating and refining these graphical models, or “causal loop diagrams”. CLD elaboration activities and model review activities, described in Table 1, provided an opportunity to assess the structure of the model for face validity and to derive insights about how session participants understood the structure of the problem. These discussions and group insights were documented by members of the facilitation team through handwritten notes and reflected via revisions to the model. Throughout the course of the sessions, this group analysis drove the development of multiple iterations of the model, all of which were documented through photographs and notes.

The session was initiated with an exercise to develop a common perspective of mental disorders to come to a shared understanding of the central concept of mental illness (Table 1). Findings illustrate the complexity of defining psychiatric and psychological disorders as well as learning disability in Dari and Pashto – two main languages used in Afghanistan - as also shown by previous work done in Afghanistan around mental health [55–58]. Dari and Pashto do not have clearly specified terms for mental illness. Session participants referred to: (i) *Dewana* which is a pejorative term, meaning “mad” or “crazy” with strong stigma associated to it; and (ii) *Rawani* which is a broad concept which includes mental illness and intellectual disability, and can commonly be described as “someone who acts like they are young”. One participant described the differences in common usage bluntly: “*Diwana is always a problem. The person causes trouble. She is not considered as normal, there is no way her condition can improve*”. Other terms referring to mental distress and anxiety such as “*asabi*” signifiying “nervousness” and “agitation” were briefly discussed as well. Previous discussions with organizational leadership and CBR workers in the context of training sessions revealed significant variation in individual conceptions of what qualifies as mental illness. Participants came to a common approximation of the English term for mental illness, termed as “Rawani” but specifying a focus on ‘severe psychological problems’ and drawing a distinction between intellectual and mental disability. The specification of terms within the modeling session allowed us to develop a model of a complex concept that did not have a clear Dari or Pashto language analog.

Between sessions and after the conclusion of the workshop, the researchers iterated a further series of revised model using Vensim PLE software. Revisions primarily focused on closing implicit feedback loops and taking decisions about how constructs could be aggregated or disaggregated to clarify meaning. All revisions were grounded in the model itself and based on notes taken by the facilitation team during the GMB session.

To examine the contributions of CBSD to an overall understanding of the dynamics of support for better access to mental healthcare for PMI, we used a framework of Levels of Community-level System Insights, proposed by Hovmand [52]. This framework provides a lens through which to understand the types of system insights gained through model building activities, from surface level descriptions of the system to deep insights about the causes of system behavior.

## Results

**Fig. 2:**
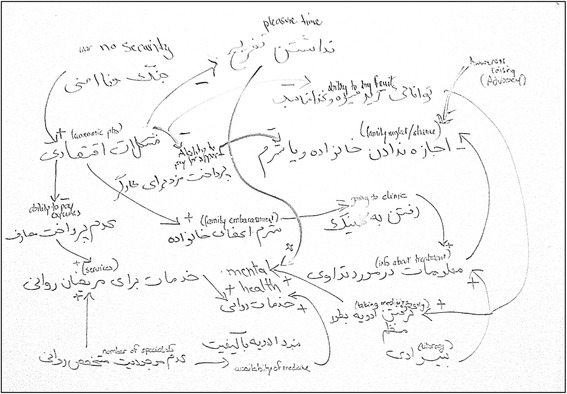
Example of a Causal Loop Diagram for exploring barriers and facilitators of service seeking for persons with mental disorders

**Fig. 3:**
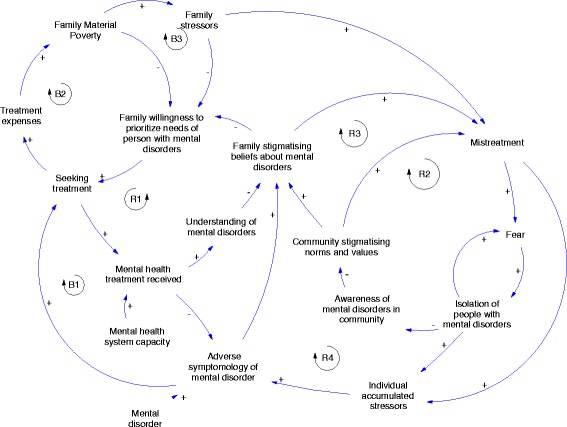
Final Causal Loop Diagram

The product of the different GMB sessions was a series of causal loop diagrams that document the evolving vision of the complex challenge of providing mental health services for people with mental illness. Figure 2 presents the preliminary CLD produced at the end of session 1, and Fig. 3 presents the revised CLD.

Causal loop diagrams (CLDs) can be read using a few key principles. Arrows, or links, represent causal relationships. The plus and minus symbols of the model indicate the polarity, or the direction of the causal relationship. The plus sign indicates a relationship that goes in the same direction, a minus sign represents an inverse relationship. For example as a family’s material poverty increases, this causes a commensurate increase in stressors on the family. The inverse is also true: if a family’s material poverty reduces, that reduces family stressors. This is a positive relationship. The relationship between family stressors and family willingness to prioritize the needs of persons with mental disorders in Fig. 3 represents an inverse relationship. As family stressors increase, this causes a decrease in families’ willingness and ability to prioritize the healthcare and other needs of a family member with mental disorders. The opposite of course is true. As family stressors decrease, they have more capacity and willingness to prioritize the needs of a family member with mental disorders.

The revised model in Fig. 3 contains multiple interacting feedback loops. Table 2 summarizes the major feedback loops that constitute a hypothesis of how system components interact to generate a persistently low rate of service utilization by people with mental disorders.

The first balancing loop (B1) shows in Table 3 that if persons with mental illness seek more treatment, adverse symptoms might be reduced, encouraging them to seek more treatment and show more medical compliance. (B2) displays the vicious cycle between poverty and mental disorders: people are poor and cannot afford to spend even small amount on medical care for the PMI, making the situation of scarcity of mental care within the BPHS (supposedly free) even more daring for those families. (B3) links this relationship between treatment needs associated with mental illness and poverty to the stressors caused by the risk of falling deeper into poverty if the family has to spend resources for the medical needs of the PMI.

**Table 3:** Important feedback loops found in the final Causal Loop Diagram model

Loop	Name	Description
B1	“Care seeking”	Adverse symptoms of mental disorders lead a person with mental disorders to seek mental health treatment, which increases the treatment she has received. This treatment reduces adverse symptoms of the mental disorders.
B2	“Treatment expenses”	Seeking care incurs treatment expenses, which increase the material poverty of the family. This material poverty reduces family’s overall ability to pay for treatment expenses, thereby reducing their willingness to seek treatment for the family member with mental disorders.
B3	“Stress of material poverty”	Seeking care incurs treatment expenses, which increase the material poverty of the family. Material poverty increases family stressors, which decreases a family’s willingness to prioritize the needs of the family member with mental disorders, resulting in that family member seeking less treatment.
R1	“Impact of treatment on family stigma”	Treatment for mental health disorder improves understanding of mental disorders by both family and the individual, which reduces stigmatizing beliefs among the family and increases their prioritization of the needs of the family member with mental disorder.
R2	“Isolation and community stigma”	Stigmatizing community norms and values create a high rate of mistreatment of people with mental disorders, which creates fear for both individuals and families and results in isolation of people with mental disorders. Isolation translates in less awareness of mental disorders in community because there is little contact with people with mental disorders, which in turn reinforces stigmatization in the community.
R3	“Community stigma driving family stigma”	Stigmatizing norms in the community also have an impact on increasing family stigmatizing beliefs about mental disorders, which in turn leads to mistreatment within the home. Mistreatment increases fear and isolation, which results in less awareness of mental disorders within the community and increases stigmatizing norms in the community, which reinforce family stigmatizing beliefs.
R4	Mistreatment and adverse symptomology of mental disorder	Mistreatment of persons with mental disorder is a source of individual stressors that negatively impacts the symptomatology of mental disorders. An increase in adverse symptomology of mental disorders reinforce family stigmatizing beliefs and increase mistreatment.

The four reinforcing feedback loop demonstrate the many ways in which public stigma impacts the wellbeing of PMI. (R1) indicates that as understanding of mental illness becomes more common, families’ stigmatizing beliefs about mental illness lessen. Again the inverse is true. As understanding is reduced, stigmatizing beliefs increase. The second reinforcing loop illustrates a worrying effect of stigma: mistreatment of PMI. As norms and values reflect increasingly prejudice and discrimination of PMI, likelihood of them being mistreated raises, resulting in fear and isolation from the community to prevent mistreatment. The third reinforcing loop shows that as stigmatizing norms and values are more prevalent among the community, so are stigmatizing beliefs about mental illness. The opposite is true; as community stigmatizing norms decrease family stigmatizing beliefs also decrease. Finally, (R4) shows how stigma, by fueling practices of various forms of mistreatment (use of bad language and bullying, harassment, physical violence), has a negative effect of the mental state of the PMI which in turn influences negatively beliefs and behaviors towards PMI.

At the close of the sessions the facilitators initiated a discussion about possible leverage points to change the current dynamic and introduce interventions in the existing system to improve access to mental healthcare for PMI.

One participant argued, “the easiest intervention is awareness with a lot of positive outcomes. If CBR workers inform people through home training and community based sensitization intervention, this can reduce existing fear. We probably need more psychologists to train our CBR workers and scale up our awareness program. And the EPHS/BPHS system lacks resources to pay those professionals as well.” Another participant added: “CBR workers can address persons with mental illness (rawani) to health clinics or hospitals within the BPHS and EPHS. It is an important role because they are the only one doing outreach to families”. Another one mentioned that the family is the first place to carry out awareness and sensitization: “If the family does not accept the mental illness how can the wider community accept it?”. Participants agree that awareness can have leverage on different aspects of the problems. “Once we identify a person with mental illness, we can approach the family, induce some positive behaviors such as avoiding naming the person *dewana*, promoting participation in family activities, involvement in ceremonies and encourage interaction with the community”. “If we manage to reduce such behaviors, we definitely improve the wellbeing of the person and her family. […] In other words, more acceptance, more happiness”. “If a community is aware of the issue of mental illness, then she can also lobby the government to provide services. If the government is pressured to intervene, it will pledge resources to hire psychologist in healthcare facilities that in turn will be able to conduct training for CBR and health workers that go in the communities to identify persons with mental illness. This model exists for midwife which requires a 2 years training. But obviously this has a cost and maybe we will have to start modestly with short term trainings”. Another added: “If we explain to the family that the condition can improve, then the family will be encouraged to seek for treatment. In Mazar for instance, we have 2 or 3 specialized doctors. If the family is not sensitized to mental illness, and the CBR worker asked them to consult one of these psychiatrists, the family will not take the person with mental illness because she is afraid of people saying she is *dewana.* People are difficult to convince *but* the first step is changing attitudes”. Another one mentioned: “the causal loop diagram shows a clear path for CBR intervention. CBR workers can work with the community to reduce stigma. Working in the community, they have a privileged access to Mullahs, teachers and elders (*Shurah* or village assembly members) that they can influence and convince to spread a message of inclusion and tolerance”.

## Discussion

The CBSD model developed collaboratively between CBR workers and team leaders and researchers provides insight into the factors that impede access to mental healthcare for persons with mental illness and what intervention could be done to change the *status quo*. CBSD establishes the causal loop relationships that explain poor access to mental healthcare and identifies points of leverage for intervention. Because the points for intervention were identified by people directly involved with the problem with support from experts facilitating the process of identification, the stakeholders may be better motivated to implement the solutions they found. In fact, our approach shares with ToC the aim of exploring solutions to complex problems using a participatory approach and ensuring stakeholders buy-in and sense of ownership [59]. In both approaches, solutions to address the problem take into account the context, in particular existing needs, difficulties such as power relations, barriers to intervention and possible remedies to problems [60]. Yet, the CBSD approach differs from a ToC approach in the method used. The ToC works backward from defining in partnership the intended impact – e.g. improve access to care, to determine required intermediate and short-term outcomes to achieve the aim, and the related indicators associated with each outcome [59, 61]. Furthermore, the CBSD approach does not assume ex ante the adoption of any component of a mental health care package as in the example of the Programme for Implementing Mental Health Care (PRIME) program using a ToC approach [8] but engage stakeholders and let them determine first the system and its components and identify leverage points for intervention. As a result, the CBSD approach is a tool that can be used before a ToC is elaborated. The CBSD approach gives a voice to stakeholders who can share their views of a problem, come up with solutions about what could be done to address them, elements that can be embedded in a ToC.

### Components of the system

Surface level system insights that emerge from the model include the composition and interaction between individual, family, and community level variables. The model describes the broad connections between family economic situations, mental healthcare seeking, and mistreatment in the community and family. The scope of these components represents a vision of care-seeking that is centered on family decision making and is contingent upon both the availability of such healthcare and the social and economic environment of the family. A dynamic hypothesis that emerges from this model is that the interaction of economic burdens of generalized poverty and treatment expenses interact with cultural and social stigmatizing beliefs, in the context of limited clinical or other treatment support, to perpetuate low access of any form of care for people with mental illness. This interaction of feedback loops describes a situation in which, even if clinical mental healthcare capacity were to be introduced, community stigma and economic forces would still represent significant barriers to access. This simple visual representation connects a number of important insights that have been shown separately by different studies: limited resources are available for mental healthcare services in Afghanistan despite original initiatives [4, 43, 44], that poverty plays a role in discouraging mental healthcare seeking behaviors [62] and the importance of stigma associated to mental illness [57].

As important as the content of the model are the concepts that are highlighted and left out by the participants in the session, in other words how members of a system think about their system. Specifically, participants operated with an understanding that mental illness is something that is caused by outside or unknown forces. There is an additional assumption that mental illness is treatable through clinical support, shown by the link that treatment received reduces adverse symptomology. Implicit in this mental health treatment variable is a vision of treatment that is primarily psychiatric. There was little discussion of any form of psychosocial counseling as a response to mental illness or its symptoms. Additionally, the assumptions of the model were that treatment primarily occurred through formal clinical mechanisms, though there was discussion of a role for trained outreach workers. This perspective suggests that any intervention associating medical treatment and psychosocial services will require information and sensitization of non-specialist health and rehabilitation workers to get their buy-in and participation.

### Points of entry for intervention

Finally, the model reveals a number of potential entry points for programmatic or community intervention to address low service receipt by PMI. Participants mentioned awareness of families and communities of the needs and rights of PMI as a potential leverage point. They argued that CBR workers are already experienced in reaching out to the community in villages where the CBR program is taking place through sensitization campaigns to promote acceptance and change attitudes towards people with disabilities generally. A sensitization effort to reach out to families (the “Understanding of mental disorders” variable) and communities (“Awareness of mental disorders in community”) would potentially have significant impact on the adverse symptomology of mental illness through reductions in stigma and in mistreatment, as well as through increased willingness to support treatment seeking for affected family members. Participants in the model building sessions discussed that such sensitization programs could work through multiple avenues: through direct face-to-face outreach with families of PMI; sessions with organizations of persons with disabilities (DPOs); and participating in community events within schools, mosques and during sessions of village *shuras (*committees of elders*)* meetings. Similarly, studies using a ToC approach identified the need for mental health awareness raising and engaging with PMI and their families as important activities in other low-income contexts [63].

Other points of programmatic entry into this system were identified as potentially valuable, but not strictly within the purview of the CBR Program. Investments in developing the capacity of the mental healthcare system through the development of new training expertise within Afghanistan for psychiatrists, psychologists, and potentially social workers could be another avenue for NGO involvement. Such intervention has been pioneered by Healthnet TPO in Afghanistan [4]. Other studies have shown elsewhere the need for specialized mental health professionals to drive the process of developing and integrating mental healthcare as part of the primary healthcare system [64, 65]. Finally, promotion of family livelihood strategies would affect the overall family context, which is argued to have a central, if indirect role in the experience and support of PMI. This finding reinforces emerging literature demonstrating the association that exists between poverty, stigma and mental illness in low-income countries [66].

### Limitations

Our study is the first example of the use of community based system dynamics looking at mental illness in a conflict setting. Because of the new context, multiple challenges in the design and facilitation of the sessions had to be addressed, which are reported here. One of the strengths of this approach is the ability to make explicit the subjectivities of individuals who are building the model. This perspective of participants who are embedded in the system allows for insights into interconnections and dependencies that may not be apparent from an external view. This strength also argues for caution: this subjectivity comes with biases and limitations of knowledge that may challenge the validity of findings. As the sessions were intended to be a rapid exploration of the initial findings of the impact evaluation study, the scope of the study was limited to participation by CBR workers. Without the voices of people with mental illness included in the study, there are clear biases in the understanding of mental illness. For example, discussions of mental health treatment were primarily focused on a vision of treatment that is primarily psychiatric. There was little discussion of any form of psychosocial counseling as a response to severe mental disorders or its symptoms. The choice to include only CBR workers was based on both logistical feasibility as well as concern for the appropriateness of engaging vulnerable stakeholders in an exploratory study. Further extension of the method to include people with mental illness would be a logical and desirable next step, but would require significant consideration of the framing of the approach and composition of facilitation team. Additionally, generalizing findings to whole organization based on the vision of a few stakeholders may jeopardize validity. Replication and triangulation through multiple sessions with diverse stakeholder groups would be necessary to strengthen findings. The co-development of a model represents a deeper level of engagement with the problem than conventional qualitative research methods such as focus groups, yet, convergence of opinions by participants in a system model does not necessarily translate to intention or capacity for action. As with any participatory method, the CBSD approach requires involvement of organizational leadership to implement findings and recommendations. Finally, the role of the outside facilitators cannot be ignored. The identification of the problem in this study stems from the results of a partnership with academic researchers who have experience in an Afghan context. The resulting model is a negotiation between facilitators’ prompts and participants’ understandings and perspectives. Neither would achieve the outcomes on its own.

### Implications

Our study demonstrates that CBSD methods can provide an effective tool to elicit a common vision on the complex/messy problem of access to care for PMI and identify shared potential strategies for intervention in line with the goals of the WHO’s Mental Health Action Plan 2013–2020 [67]. The process and the resulting model showed that: (i) a multiple stakeholder groups can analyze complex causal loops that impede access to care for PMI and provide ideas for intervention; (ii) a successful facilitation process preserves the vision and perspectives of participants while reaching a common understanding of unmet mental healthcare needs; (iii) a roadmap to intervention shared by various stakeholders involved in the program can be delineated with limited input of expert knowledge.

The issue of lack of access to mental health services in low-income countries is the subject of growing research and literature particularly around the need of effective interventions in context of limited financial and professional resources [68–70]. An important issue that remains to be adequately addressed is the role of stigma as a strong driver of discrimination of PMI resulting in exclusion from treatment but also from employment and community participation [66]. Such a process of exclusion results in poor self-esteem and internalized stigma, material poverty for the person and her family and deepening and mental suffering as underlined by CBSD participants [71, 72]. These dynamics articulated in the literature were elaborated over the course of only a few sessions through the complex interactions of feedback loops. They suggested that an appropriate strategy must address community and families’ perception of mental disorders to reduce stigma and barriers to seeking outside support. Participants identified the conditions for expanding the current program to address the needs of PMI: revising organizational priorities, building staff expertise and increasing in-country training capacity in psychiatry and psychology. Our study demonstrates that the CBSD modeling process can elicit these relationships with minimal expert input. This suggest that endogenous expertise – i.e. knowledge of the people involved in the system itself – may be adequate to frame a sophisticated argument about the messy problem of CBR access for people with mental illness.

## Conclusion

In a context of limited resources, the CBSD approach suggests a different path for program planning and eventually evaluation. The originality of the problem solving approach described in our study is that it is driven by people embedded within the system. It can generate robust sophisticated results with actionable policy recommendations building on the knowledge and expertise of participants.

This approach offers a new collaboration framework that privileges the knowledge of people involved in the system and focuses on outcomes that address the needs of communities. The process of community based system dynamics can provide a window for organizational reflection and the opportunity to build a common vision and momentum for action. This is particularly valuable for messy and neglected problems such as mental illness for which needs for intervention are still considerable in low-income settings.

## Abbreviations

CBR, community based rehabilitation; CBRW, community-based rehabilitation worker; CBSD, community-based system dynamics; CLD, causal loop diagram; DPO, Disabled Persons Organization; GMB, Group Model Building; NGO, Non Governmental Organization
